# Crystal structure of FadD32, an enzyme essential for mycolic acid biosynthesis in mycobacteria

**DOI:** 10.1038/srep15493

**Published:** 2015-12-02

**Authors:** Wenjuan Li, Shoujin Gu, Joy Fleming, Lijun Bi

**Affiliations:** 1 Key Laboratory of RNA Biology, Institute of Biophysics, Chinese Academy of Sciences, Beijing, China; 2 University of Chinese Academy of Sciences, Beijing, China

## Abstract

Fatty acid degradation protein D32 (FadD32), an enzyme required for mycolic acid biosynthesis and essential for mycobacterial growth, has recently been identified as a valid and promising target for anti-tuberculosis drug development. Here we report the crystal structures of *Mycobacterium smegmatis* FadD32 in the apo and ATP-bound states at 2.4 Å and 2.25 Å resolution, respectively. FadD32 consists of two globular domains connected by a flexible linker. ATP binds in a cleft at the interface between the N- and C-terminal domains and its binding induces significant local conformational changes in FadD32. The binding sites of meromycolic acid and phosphopantetheine are identified by structural comparison with other members of the adenylating enzyme superfamily. These results will improve our understanding of the catalytic mechanism of FadD32 and help in the design of inhibitors of this essential enzyme.

Tuberculosis (TB) is an infectious disease caused by the bacillus *Mycobacterium tuberculosis*. This ancient disease remains a major global health problem; one third of the world’s population is estimated to be latently infected with *M. tuberculosis*, and approximately 9 million people developed TB and 1.5 million died from this disease in 2013^1^. The continuing emergence of multidrug-resistant, extensively drug-resistant and totally drug-resistant *M*. *tuberculosis* severely threatens global TB control^2^,^3^ and development of new anti-TB drugs is therefore urgently required^4^. Targeting cell wall biogenesis in *M. tuberculosis* for drug development has proved to be very promising^5^. The mycobacterial cell wall is comprised of three covalently linked macromolecules: peptidoglycan, arabinogalactan, and mycolic acids^6^. Recently, several proteins related to mycobacterial cell wall biogenesis have been validated as new drug targets, including the L,D-transpeptidase Ldt_Mt2_ involved in peptidoglycan biosynthesis^7^, the decaprenylphosphoryl-β-D-ribose 2′-epimerase DprE1 involved in arabinogalactan biosynthesis^8^, and fatty acid degradation protein D32 (FadD32), polyketide synthase PKS13 and trehalose monomycolate transporter Mmpl3, all of which are involved in mycolic acid biosynthesis^9^,^10^,^11^.

FadD32 is one of the 34 FadD proteins of *M. tuberculosis* annotated as putative fatty acyl-CoA synthetases^12^, and its role in mycolic acid biosynthesis is to activate C_48_–C_64_ meromycolic acid for condensation by PKS13^13^. Gene knockdown experiments have demonstrated that FadD32 inhibition can severely compromise the growth of *M. tuberculosis* both *in vitro* and inside macrophages^14^. FadD32 belongs to the adenylating enzyme superfamily that includes acyl-CoA synthetases, the adenylation domains of nonribosomal peptide synthetases, and firefly luciferases. These enzymes consist of two domains and catalyze two half-reactions: an adenylate-forming reaction which leads to formation of acyl-AMP from a carboxylate substrate and ATP, and a thioester-forming reaction which leads to formation of acyl-CoA from acyl-AMP and CoA or formation of acyl-ACP from acyl-AMP and ACP^15^. Although FadD32 was initially found to catalyze only the first half-reaction and was named fatty acyl-AMP ligase (FAAL)^12^, later evidence demonstrated that it is a bifunctional enzyme: during mycolic acid biosynthesis FadD32 catalyzes both the formation of meromycoloyl-AMP from meromycolic acid and ATP and the subsequent acyl chain transfer from meromycoloyl-AMP to the phosphopantetheinyl arm of the N-terminal ACP domain of PKS13^16^,^17^. Surprisingly, structural and biochemical studies on *M. tuberculosis* FAAL28 (or FadD28) suggest that FAALs (including FadD32) cannot catalyze the second half-reaction because of an insertion motif that hinders rotation of their C-terminal domains^18^. In addition, the FAAL28 structure only contains an N-terminal domain, and so the catalytic mechanism of FAALs remains elusive. As FadD32 is a promising drug target, two groups have developed high-throughput screening methods to identify its inhibitors^19^,^20^,^21^. One set of inhibitors are 4,6-diaryl-5,7-dimethyl coumarin derivatives, which have activity comparable with that of isoniazid (a first-line anti-TB drug) in animal models of TB. Here, we have determined the structure of FadD32 in the apo and ATP-bound states in order to provide insights into FadD32 substrate recognition and catalysis and thus assist in the design of new inhibitors of FadD32.

## Results and Discussion

### Overall structure of FadD32

Full-length FadD32 from *M. tuberculosis* (MtFadD32) has been reported to be recalcitrant to crystallization^22^. As our own initial studies indicated that MtFad32 tended to aggregate, we decided to crystallize FadD32 from *M. smegmatis* (MsFadD32), which has 74% sequence identity to MtFadD32 (Supplementary Fig. 1). We expressed full-length MsFadD32 and its N-terminal domain in *Escherichia coli* and purified the protein to homogeneity (Supplementary Fig. 2). To detect the interaction between FadD32 and ATP, we performed isothermal titration calorimetry (ITC) experiments. ITC results showed that ATP bound to full-length MsFadD32 with a K_d_ value of approximately 36 μM, while AMP (control) did not bind to the protein (Supplementary Fig. 3a,b); Moreover, the N-terminal domain of MsFadD32 (N-MsFadD32) alone could not bind ATP (Supplementary Fig. 3c). Although N-MsFadD32 could be easily crystallized, we could only obtain crystals of full-length MsFadD32 by co-crystallizing the SUMO fusion protein and ATP. We determined the structures of apo N-MsFadD32 and ATP-bound MsFadD32 at 2.4 Å and 2.25 Å resolution by molecular replacement. We observed one molecule of MsFadD32 and one molecule of SUMO in the asymmetric unit of the crystal structure of ATP-bound MsFadD32, with a critical crystal contact mediated by SUMO (Supplementary Fig. 4). Data collection and refinement statistics are shown in Table 1.

**Figure 1:**
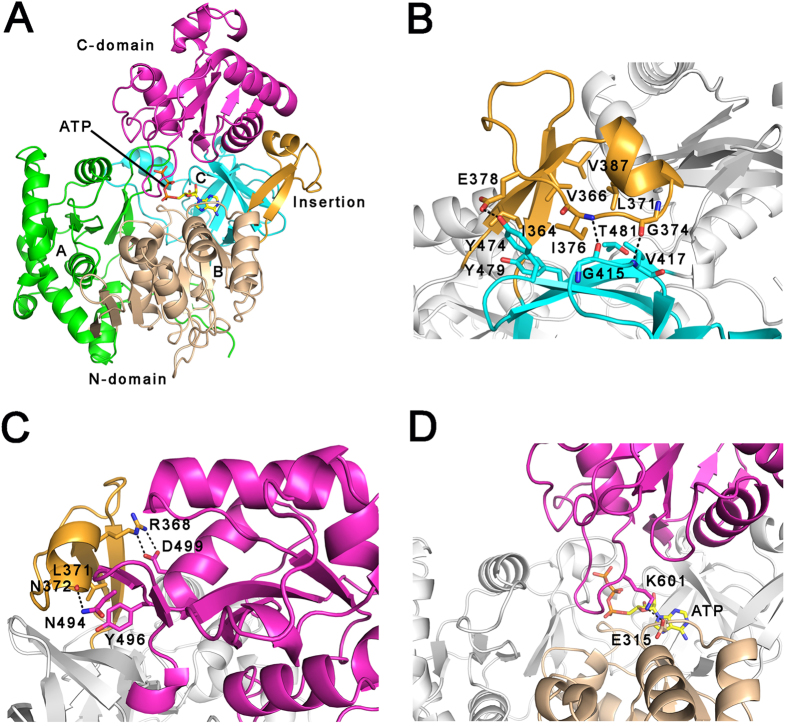
Overall structure of ATP-bound MsFadD32. (**A**) Ribbon representation of the MsFadD32 structure. The three subdomains of the N-terminal domain are colored green, wheat, and cyan, respectively. The insertion motif and C-terminal domain are colored orange and magenta, respectively. ATP is shown as yellow sticks. (**B**) The interactions between the insertion motif and N-terminal subdomain C. (**C**) The interactions between the insertion motif and C-terminal domain. (**D**) The ATP-bound MsFadD32 adopts an atypical adenylate-forming conformation. In (**C**–**D**), the residues involved are shown as sticks and the hydrogen bonds are shown as dashed lines.

The overall structure of MsFadD32 consists of two distinct domains, a large N-terminal domain comprising residues 1–483 and a smaller C-terminal domain comprising residues 484–630. The N-terminal domain can be further divided into three subdomains (A, B, and C) and an insertion motif that is located between subdomains B and C. Subdomains A and B each have a central β-sheet that is flanked by α-helices on both sides. The two subdomains juxtapose to form a five-layered αβαβα structure. Subdomain C contains a distorted six-stranded β-barrel and a small α-helix. The C-terminal domain is composed of a three-stranded β-sheet surrounded by four α-helices, and an additional small two-stranded β-sheet (Fig. 1a). Consistent with previous observations in *E. coli* FAAL (EcFAAL)^23^, the insertion motif interacts with both N- and C-terminal domains in MsFadD32. The interactions between the insertion motif and N-terminal subdomain C contain extensive hydrophobic interactions and three hydrogen bonds (Fig. 1b). These hydrophobic interactions were reported to be present in all FAALs and play an important role in anchoring the insertion motif to the N-terminal domain^24^. The insertion motif interacts with the C-terminal domain through two charge-stabilized hydrogen bonds between Arg368 and Asp499, hydrophobic interactions between Leu371 and Tyr496, and a hydrogen bond between the main-chain carbonyl oxygen of Asn372 and the side-chain nitrogen of Asn494 (Fig. 1c). These interactions likely exist in all FadD32 proteins of the *Corynebacterineae* as the residues involved are highly conserved (Supplementary Fig. 1).

Structural comparisons with other adenylating enzymes reveal that ATP-bound MsFadD32 exists in the adenylate-forming conformation. However, the invariant Lys601 in motif A10 (Supplementary Fig. 1) located in the C-terminal domain does not point into the active site and interact with ATP but forms a charge-stabilized hydrogen bond with Glu315 located in the N-terminal subdomain B (Fig. 1d). We postulate that upon binding of its other substrate, meromycolic acid, Lys601 will be orientated into the active site and interact with both ATP and meromycolate, thereby forming the true adenylate-forming conformation observed in the phenylalanine-activating subunit of gramicidin synthetase 1 (PheA)^25^ and other adenylating enzymes^15^.

### ATP-binding site

**Figure 2:**
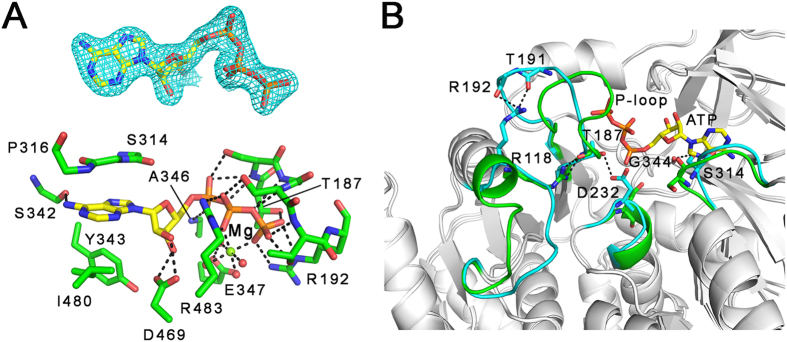
ATP binding and conformational changes in MsFadD32. (**A**) The binding environment for ATP. Top panel: the 2*Fo – Fc* electron density (contoured at 1.5σ) is shown for ATP. Bottom panel: the Mg^2+^ ion between the β- and γ-phosphates of ATP is shown as a green sphere, and the four surrounding water molecules are shown as red spheres. (**B**) ATP binding-induced conformational changes in MsFadD32. The fragments of apo and ATP-bound MsFadD32 involved in conformational changes are colored cyan and green, respectively.

In the MsFadD32-ATP complex, ATP sits in a cleft at the interface between the N- and C-terminal domains (Fig. 2a), and the residues involved in ATP binding are all from the large N-terminal domain. The adenine moiety of ATP is sandwiched between the side chains of Tyr343 and Ile480 on one side and the main-chain atoms of Ser314–Pro316 on the other. The adenine ring stacks against Tyr343 and is stabilized by a hydrogen bond between its N6 amino group and the main-chain carbonyl oxygen of Ser342. The ribose moiety of ATP is anchored by two hydrogen bonds between its two hydroxyls and the carboxylate of Asp469, an invariant residue among all adenylating enzymes^15^. Mutation of the equivalent residue in FadD13 (D382A) has been shown to significantly affect enzyme activity^26^. The three phosphate groups of ATP are held in place mainly by the P-loop^27^ residues (Thr187–Arg192 of motif A3 (Supplementary Fig. 1)). In particular, the guanidinyl group of Arg192, a residue conserved in all FadD32 proteins in the *Corynebacterineae* (Supplementary Fig. 1), forms bidentate hydrogen bonds with the γ-phosphate. In addition, the main-chain amide nitrogen of Ala346 forms a hydrogen bond with the α-phosphate, and the guanidinyl group of Arg483 located in the interdomain hinge region forms bidentate hydrogen bonds with the β-phosphate. The Mg^2+^ ion, which bridges the β- and γ-phosphates, is coordinated by two oxygen atoms from the β- and γ-phosphates and four water molecules. Two of the four water molecules are stabilized by forming hydrogen bonds with the carboxylate of the invariant Glu347^15^.

### ATP binding-induced conformational changes

Superposition of the structures of the apo and ATP-bound MsFadD32 revealed conformational changes within the N-terminal domain. Firstly, to accommodate the adenine moiety of ATP, the side-chain hydroxyl of Ser314, which forms a hydrogen bond with the main-chain carbonyl oxygen of Gly344 in the apo state, rotates 180°. Secondly, the side-chain carboxylate of Asp232, which forms a hydrogen bond with the side-chain hydroxyl of Thr187 in the apo state, rotates approximately 90° to make room for the α-phosphate of ATP. Thirdly, the P-loop moves a relatively large distance (5–7 Å) to stabilize the three phosphate groups of ATP. Of note, the conformational change in the P-loop is accompanied by the extension of a neighboring α-helix where Arg118, which in the apo state forms hydrogen bonds with Thr191 and Arg192, forms hydrogen bonds with Thr187 in the ATP-bound state (Fig. 2b).

### Meromycolate-binding site

**Figure 3:**
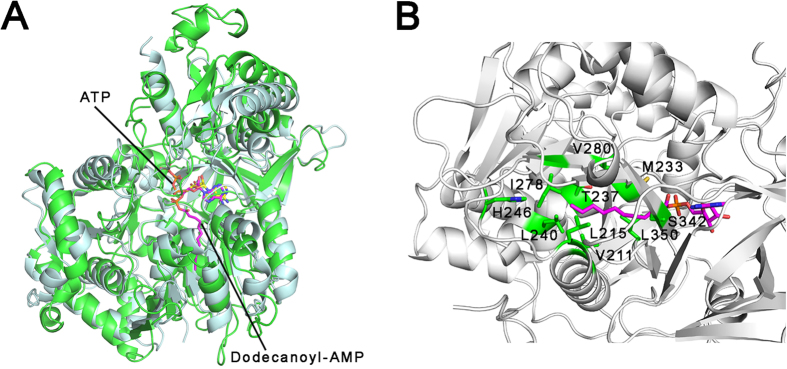
Meromycolate-binding site. (**A**) Structural superposition of ATP-bound MsFadD32 (green) and dodecanoyl-AMP-bound EcFAAL (pale cyan). Dodecanoyl-AMP is shown as a magenta stick. (**B**) The hydrophobic tunnel for meromycolate binding. The residues comprising the tunnel are shown as green sticks.

There is a hydrophobic tunnel in the ATP-bound MsFadD32 structure at the opposite side of the phosphate tail of ATP. We found that, upon superposition of the structures of ATP-bound MsFadD32 and dodecanoyl-AMP-bound EcFAAL, the AMP components of ATP and dodecanoyl-AMP nearly overlap and the C_12_ tail of dodecanoyl-AMP points into the hydrophobic tunnel of MsFadD32 (Fig. 3a). However, unlike the huge hydrophobic channel present in the human Ag-presenting molecule CD1b^28^, the hydrophobic tunnel in MsFadD32 is clearly too small to accommodate the very long (C_48_–C_64_) meromycolic acid (Fig. 3b). We suggest that the hydrocarbon tail of meromycolate may protrude from the bottom of the hydrophobic tunnel and enter into another hydrophobic tunnel present in a partner protein of FadD32, such as PKS13. The direct interaction between FadD32 and PKS13 has been implicated in previous biochemical studies^16^. Consistent with this hypothesis, we found that MsFadD32 and MsPKS13 coeluted from a gel filtration column (Supplementary Fig. 5).

### Phosphopantetheine-binding site

**Figure 4:**
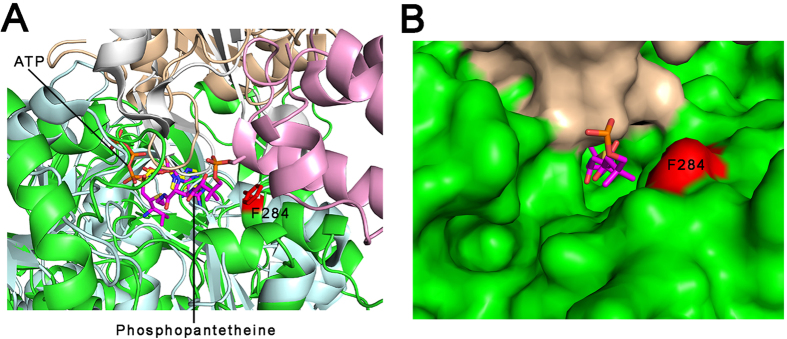
Phosphopantetheine-binding site. (**A**) Structural superposition of ATP-bound MsFadD32 and adenylate analog- and phosphopantetheine-bound PA1221 protein. The N- and C-terminal domains of MsFadD32 are colored green and wheat, respectively. The N- and C-terminal subdomains and the peptidyl carrier protein domain of PA1221 are colored pale cyan, white and pink, respectively. The covalently-connected adenylate analog and phosphopantetheine are shown as magenta sticks. Phe284 of MsFadD32 is shown as a red stick. (**B**) Surface representation of the two domains of MsFadD32 showing the phosphopantetheine-binding tunnel.

According to previous biochemical and structural studies on other adenylating enzymes^15^, the phosphopantetheine-binding tunnel of MsFadD32 is expected to be present at the interface between the N- and C-terminal domains and to be created when the C-terminal domain rotates approximately 140° to form the thioester-forming conformation. Of note, the glycine residue in the A8 motif that interacts with the β-alanine moiety of the pantetheine in other adenylating enzymes^15^, i.e. Gly492, is conserved in FadD32 (Supplementary Fig. 1). In the current adenylate-forming conformation, one half of the phosphopantetheine-binding tunnel in MsFadD32 can be identified by superposing the structures of ATP-bound MsFadD32 and the adenylate analog- and phosphopantetheine-bound PA1221 protein^29^. Upon superposition, the AMP components of ATP and the adenylate analog nearly overlap and the pantetheine-binding tunnel of PA1221 corresponds well with a tunnel located at the interdomain interface of MsFadD32. Phe284 is located at the entrance of the putative pantetheine-binding tunnel of MsFadD32 (Fig. 4). A previous study has shown that mutation of the equivalent residue of MtFadD32 (F291L) results in resistance to 4,6-diaryl-5,7-dimethyl coumarin derivative inhibitors in *M. tuberculosis*^19^. We therefore suggest that these potential drugs inhibit FadD32 activity by binding to the entrance of the phosphopantetheine-binding tunnel, thus preventing pantetheine binding, consistent with a previous finding that 4,6-diaryl-5,7-dimethyl coumarin derivatives inhibit acyl chain transfer from acyl-AMP to the phosphopantetheinyl arm of PKS13 or the mycobacterial acyl carrier protein (AcpM)^19^.

In conclusion, we have presented the first crystal structures of FadD32, an enzyme essential for mycolic acid biosynthesis in mycobacteria and identified the binding sites of its substrates, including ATP, meromycolic acid and phosphopantetheine. The full-length structure of FadD32 shows that although the insertion loop has extensive interactions with the N-terminal domain, its interaction with the C-terminal domain is not strong. We therefore suggest that upon PKS13 binding, FadD32 may undergo a conformational change, leading to weakening of the interactions between the insertion loop and the C-terminal domain and rotation of the C-terminal domain to the thioester-forming conformation. Further mutational studies on the residues mediating the interactions between the insertion loop and the C-terminal domain will be required to study the catalytic mechanism of FadD32. In addition, the structures shed light on the mechanism of inhibition of FadD32 by 4,6-diaryl-5,7-dimethyl coumarin derivatives and lay the foundation for designing new inhibitors of this essential enzyme. We suggest that a small molecule which could bind to the interface between the insertion loop and the C-terminal domain and enhance their interactions would make a good inhibitor of FadD32 activity.

## Methods

### Protein expression and purification

The pMCSG7-N-MsFadD32 vector^30^ containing the N-terminal domain (residues 1–484) of *M. smegmatis* FadD32, pET28-SMT3-MsFadD32 vector^31^ containing SUMO-fused full-length *M. smegmatis* FadD32, and pMCSG7-MsPKS13ΔC vector containing residues 1–1042 of *M. smegmatis* PKS13 were constructed and transformed into *E. coli* BL21 (DE3) for overexpression. After IPTG induction at 289 K for 18 h, the cells were collected and resuspended in lysis buffer containing 20 mM Tris, pH 8.0, 500 mM NaCl, and 20 mM imidazole. After sonication, the cell lysate was centrifuged at 16000 rpm, 277 K, and the supernatant containing the tagged protein was applied to a nickel-affinity column pre-equilibrated with the lysis buffer. The recombinant protein was eluted with 250 mM imidazole and further purified by size-exclusion chromatography (SEC). The N-MsFadD32 protein sample was treated with TEV protease to remove the N-terminal His-tag before SEC. The resulting protein sample was >95% pure, as determined by SDS-PAGE.

### Crystallization and structure determination

Crystallization trials were performed using the sitting-drop vapor-diffusion method. The protein was concentrated to approximately 10 mg ml^-1^ and mixed in a 1:1 ratio with reservoir solution. N-MsFadD32 crystallized in 0.1 M magnesium formate, 15% PEG 3350 at 289 K. For crystallization of SUMO-fused full-length MsFadD32 at 281 K, the protein was pre-incubated with 10 mM ATP and 20 mM MgCl_2_ at 277 K for 2 h and then mixed with 0.2 M sodium malonate, 20% PEG 3350. Diffraction data were collected from flash-frozen crystals at 100 K using a Rigaku MM007-HF CCD (Saturn 944HG) diffractometer and the beamline BL17U of the Shanghai Synchrotron Radiation Facility.

All data sets were processed using HKL2000^32^. The structures of apo N-MsFadD32 and ATP-bound MsFadD32 were determined by molecular replacement using PHASER^33^ and search models derived from the N-terminal domain of MtFAAL28 (PDB ID: 3E53) and the C-terminal domain of EcFAAL (PDB ID: 3PBK). Models were built manually with COOT^34^ and refined with PHENIX^35^. The stereochemical quality of the refined structures was verified with PROCHECK^36^. Most of the residues have good geometry, and no residues were present in the disallowed regions of the Ramachandran plots. Data collection and refinement statistics are presented in Table 1. All structural representations in this paper were prepared with PyMol (DeLano Scientific).

### Isothermal titration calorimetry assay

The binding affinities between MsFadD32 and ligands were measured with an ITC200 micro-calorimeter (MicroCal). Full-length MsFadD32 and its N-terminal domain were prepared in a buffer containing 20 mM Tris, pH 8.0, 150 mM NaCl, 3 mM MgCl_2_. ATP or AMP was dissolved in the same buffer. Protein concentration in the measurement cell was 50 μM, while the ligand concentration in the titration procedure was adjusted to 600 μM. Titrations were performed at 298 K, and data were fitted to a one-binding-site model using Origin 7.0 (MicroCal).

### Gel filtration assay

A Superdex 200 column was used for gel filtration assays, and the column was pre-equilibrated with a buffer containing 20 mM Tris, pH 8.0, 150 mM NaCl. To examine the interaction between MsFadD32 and MsPKS13, SUMO-fused full-length MsFadD32 was treated with Ulp1 protease to remove the N-terminal SUMO protein and the resultant MsFadD32 was used in subsequent experiments. MsFadD32 pre-incubated with MsPKS13ΔC, and individual MsFadD32 and MsPKS13ΔC proteins, were injected into the column and eluted at a flow rate of 0.4 ml min^-1^. Peak fractions were collected and analyzed by SDS-PAGE.

## Additional Information

**Accession codes:** Coordinates and structure factors for apo N-MsFadD32 and ATP-bound MsFadD32 have been deposited in the Protein Data Bank under accession codes 5D6N and 5D6J, respectively.

**How to cite this article**: Li, W. *et al.* Crystal structure of FadD32, an enzyme essential for mycolic acid biosynthesis in mycobacteria. *Sci. Rep.* **5**, 15493; doi: 10.1038/srep15493 (2015).

## Supplementary Material

Supplementary Information

## Figures and Tables

**Table 1:** Data collection and refinement statistics.

	N-MsFadD32	ATP-MsFadD32
Data collection		
Space group	*P*2_1_2_1_2_1_	*P*4_3_2_1_2
Cell dimensions		
*a*, *b*, *c* (Å)	61.7, 78.2, 102.2	122.2, 122.2, 142.6
α, β, γ (°)	90.0, 90.0, 90.0	90.0, 90.0, 90.0
Resolution (Å)	2.40 (2.49–2.40)	2.25 (2.33–2.25)
*R*_merge_	0.128 (0.554)	0.099 (0.557)
*I*/*σI*	17.4 (4.2)	26.5 (6.2)
Completeness (%)	99.8 (99.5)	100 (100)
Redundancy	10.2 (9.4)	14.3 (14.8)
Refinement		
Resolution (Å)	25.68–2.40	29.99–2.25
No. reflections	19,858	51,768
*R*_work_/*R*_free_	0.185/0.243	0.169/0.206
No. atoms		
Protein	3,691	5,429
Ligand/ion	–/–	31/1
Water	290	482
*B*-factors (Å^2^)		
Protein	30.6	46.2
Ligand/ion	–/–	28.7/31.2
Water	31.5	45.8
R.m.s. deviations		
Bond lengths (Å)	0.002	0.005
Bond angles (°)	0.629	0.951
Ramachandran plot		
Favored (%)	97.3	98.1
Allowed (%)	2.7	1.9
Outliers (%)	0.0	0.0

Values in parentheses are for the highest-resolution shell.
