# Supplementary material for: Crystal structure of FadD32, an enzyme essential for mycolic acid biosynthesis in mycobacteria
Source: Sci Rep. 2015 Dec 2;5:15493. doi: 10.1038/srep15493 (PMC4667280; doi:10.1038/srep15493)
Supplement: Supplementary Information [file srep15493-s1.doc]

Supplementary Information for

**Crystal structure of FadD32, an enzyme essential for mycolic acid biosynthesis in mycobacteria**

Wenjuan Li^1*^, Shoujin Gu^1,2*^, Joy Fleming^1^, and Lijun Bi^1^

^1^Key Laboratory of RNA Biology, Institute of Biophysics, Chinese Academy of Sciences, Beijing, China

^2^University of Chinese Academy of Sciences, Beijing, China

**
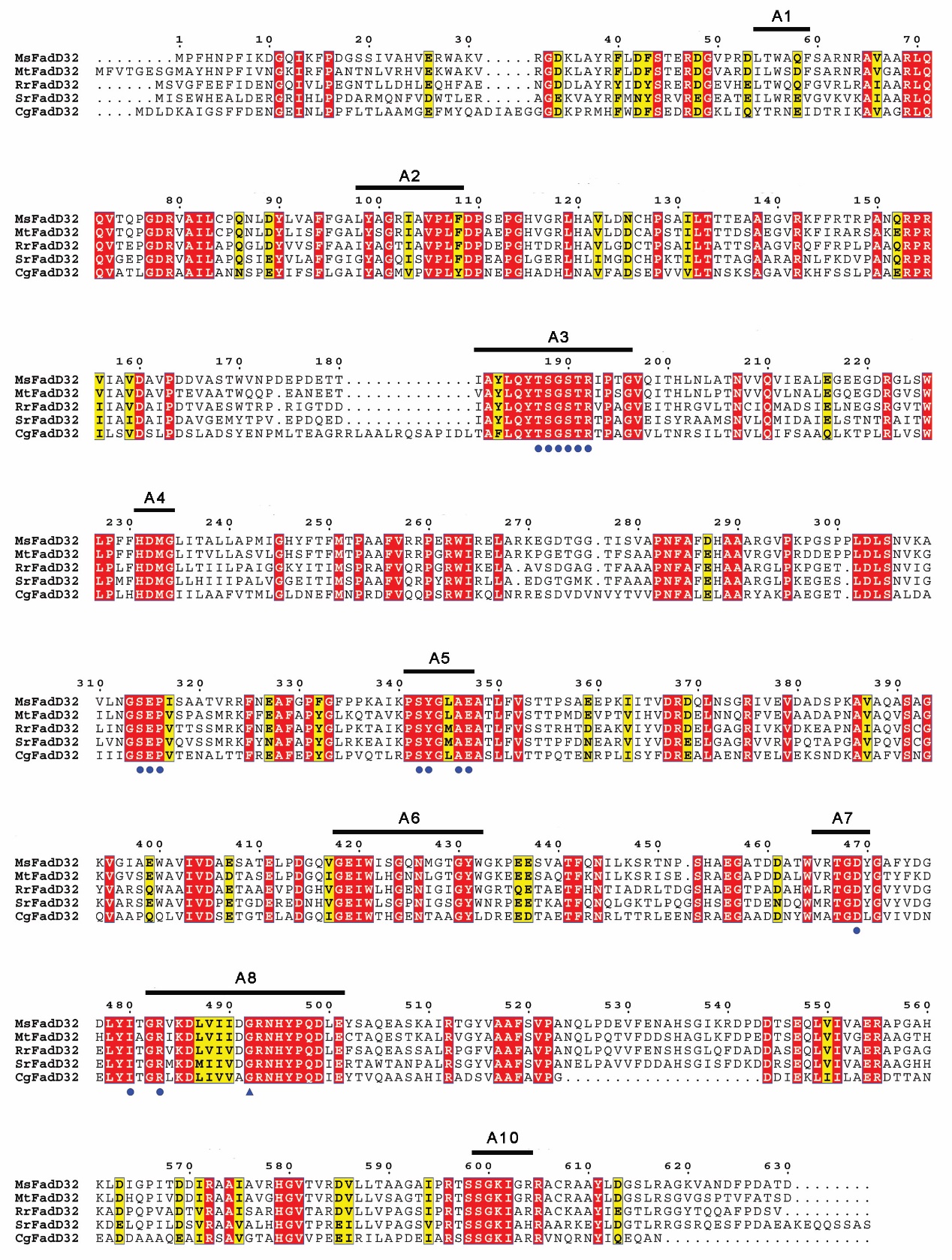
Supplementary Fig. 1 | Sequence alignment of FadD32 from different *Corynebacterineae*.** Species used for alignment were *Mycobacterium smegmatis* (Ms), *Mycobacterium tuberculosis* (Mt), *Rhodococcus ruber* (Rr), *Segniliparus rotundus* (Sr), and *Corynebacterium glutamicum* (Cg). The A1-A10 motifs conserved in ANL superfamily enzymes are indicated for FadD32 according to the defined adenylation domains of nonribosomal peptide synthetases^1^. The A9 motif, however, could not be found in FadD32 because of low conservation. Residues involved in ATP binding are marked by blue circles. The universally conserved Gly492 that interacts with phosphopantetheine is marked by a blue triangle.


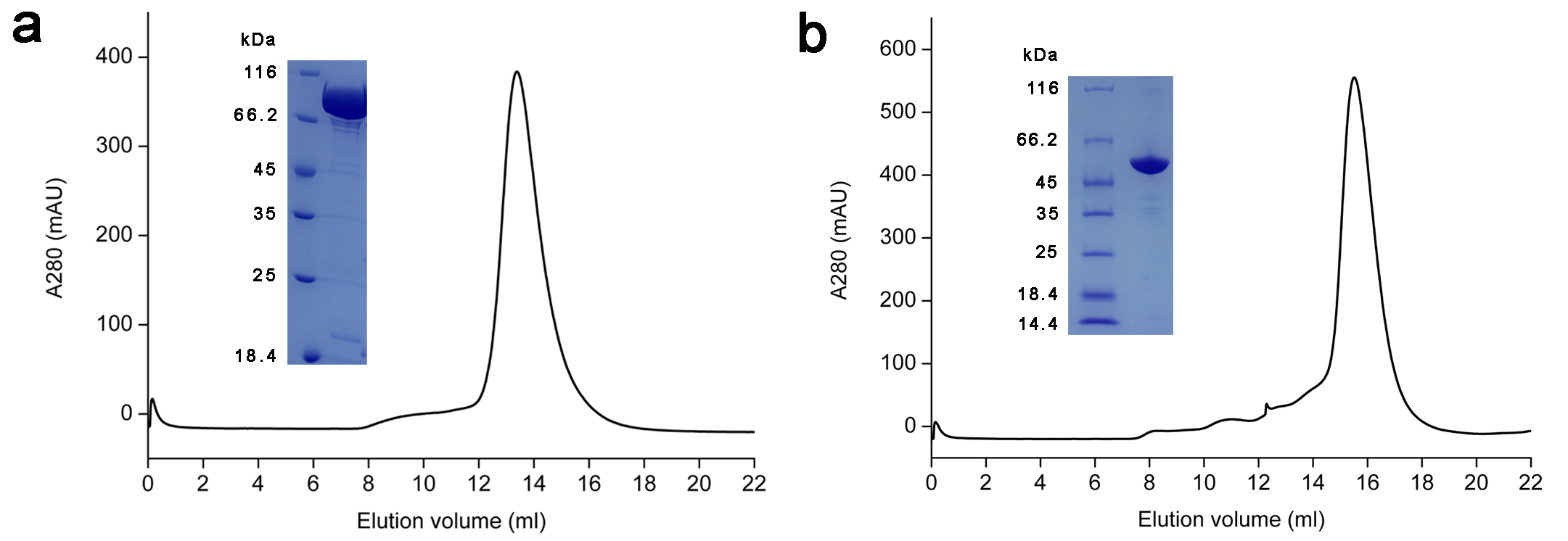


**Supplementary Fig. 2 | Purification of MsFadD32**. SUMO-fused full-length MsFadD32 (a) and the N-terminal domain of MsFadD32 (b) are homogeneous based on their gel filtration profiles and SDS-PAGE.


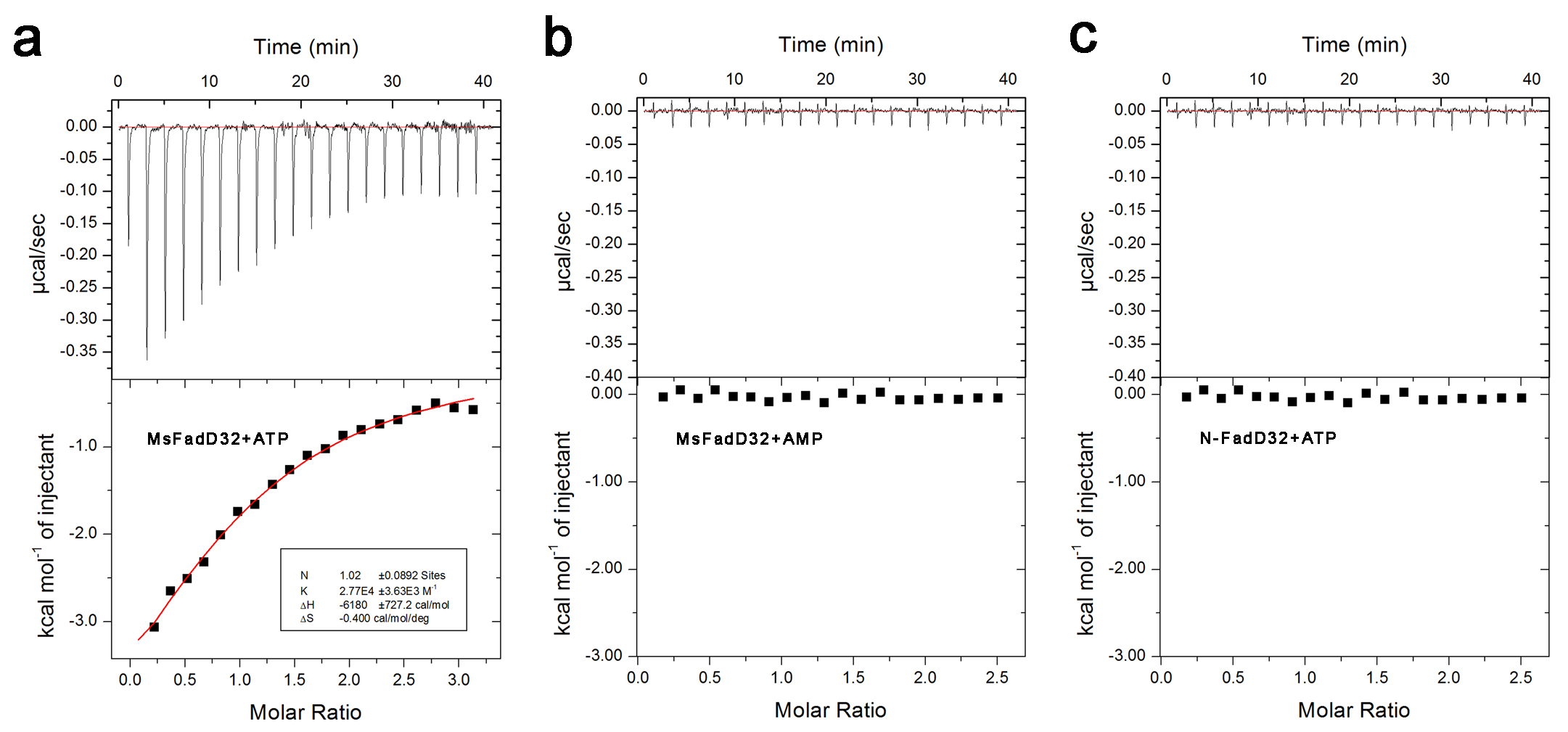


**Supplementary Fig. 3 | Detection of the interaction between FadD32 and ATP by ITC**. (a) Titration of full-length MsFadD32 with ATP. (b) Titration of full-length MsFadD32 with AMP (control). (c) Titration of the N-terminal domain of MsFadD32 with ATP.

**
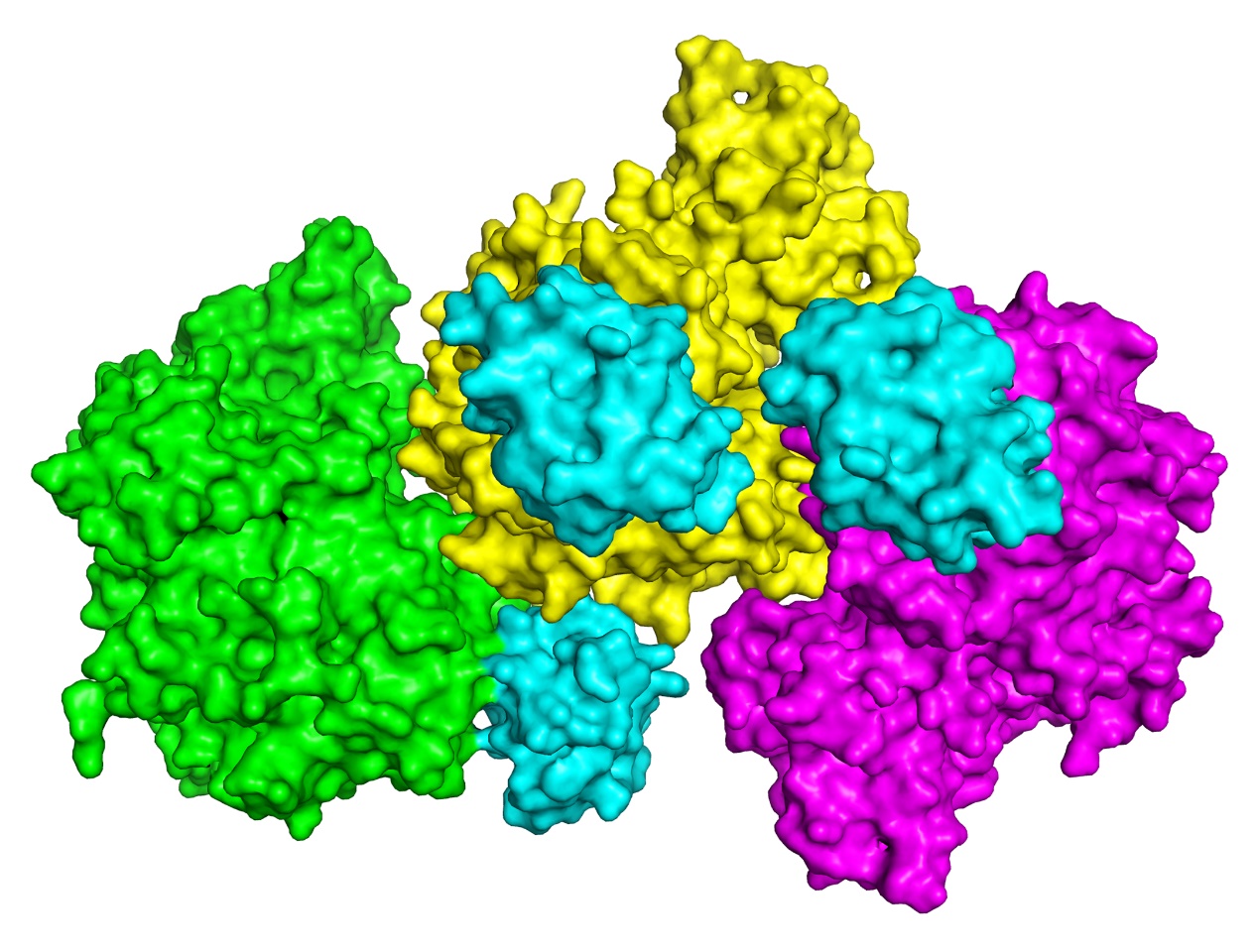
**

**Supplementary Fig. 4 | Packing of three asymmetric units in the crystal lattice.** The three MsFadD32 molecules are colored green, yellow and magenta, respectively. The SUMO molecule is colored cyan.

**
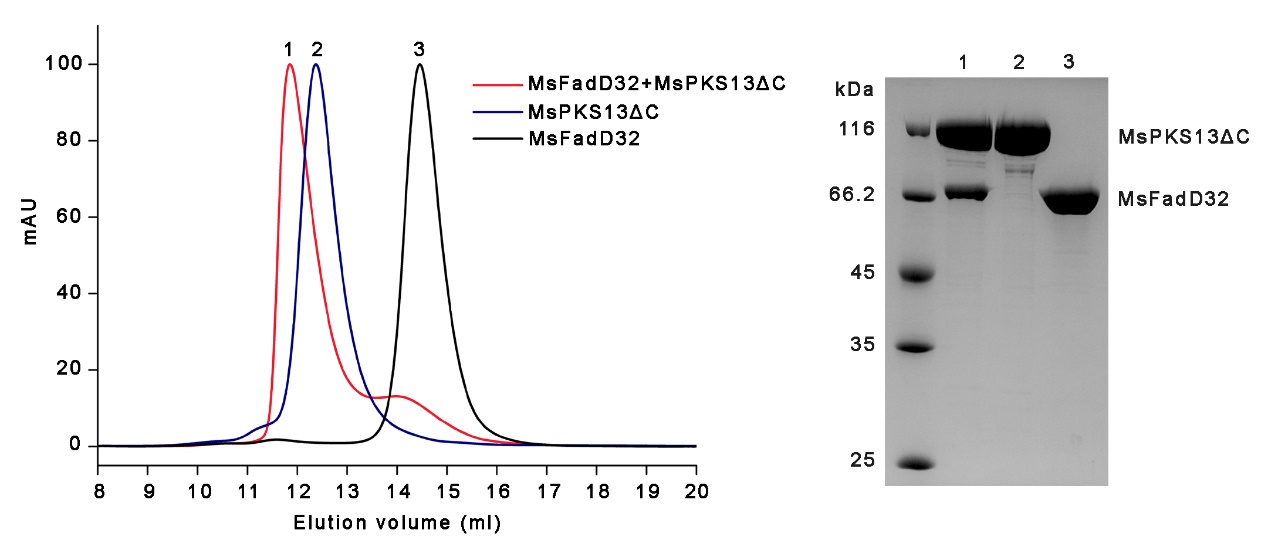
**

**Supplementary Fig. 5 | Detection of the interaction between MsFadD32 and MsPKS13 by gel filtration assays.** Left panel: elution profiles of MsFadD32 pre-incubated with MsPKS13ΔC, and MsFadD32 or MsPKS13ΔC. As full-length MsPKS13 tended to aggregate, MsPKS13ΔC (lacking C-terminal ACP and TE domains) was used for gel filtration assays. Right panel: Coomassie blue staining of the peak fractions shown on the left following SDS-PAGE.

**References**

1. Marahiel, M.A., Stachelhaus, T. & Mootz, H.D. Modular peptide synthetases involved in nonribosomal peptide synthesis*. Chem. Re***v.**97, 2651-2674 (1997).
